# Association of insulin use with LV remodeling and clinical outcomes in diabetic patients with heart failure and reduced ejection fraction: assessed by cardiac MRI

**DOI:** 10.1186/s12933-023-01944-y

**Published:** 2023-08-04

**Authors:** Ke Shi, Ge Zhang, Hang Fu, Shan Huang, Hua-Yan Xu, Yue Gao, Rui Shi, Wei-Feng Yan, Wen-Lei Qian, Yuan Li, Ying-Kun Guo, Zhi-Gang Yang

**Affiliations:** 1 Department of Radiology, Functional and Molecular Imaging Key Laboratory of Sichuan Province, West China Hospital, Sichuan University, Chengdu, Sichuan China; 2 Department of Radiology, Sichuan Cancer Center, School of Medicine, Sichuan Cancer Hospital and Institute, University of Electronic Science and Technology of China, Chengdu, Sichuan China; 3 Department of Radiology, Key Laboratory of Birth Defects and Related Diseases of Women and Children of Ministry of Education, West China Second University Hospital, Sichuan University, Chengdu, Sichuan China

**Keywords:** Heart failure with reduced ejection fraction, Insulin, Left ventricular remodeling, Contractile dysfunction

## Abstract

**Background:**

Insulin is commonly used in type 2 diabetes mellitus (T2DM) to achieve glycemic control. However, recent evidence showed that insulin use is associated with poor outcomes in the context of heart failure (HF). Since heart failure with reduced ejection fraction (HFrEF) accounts for approximately 50% of cases in the general HF population, we aimed to evaluate the effect of insulin treatment on left ventricular (LV) remodeling and contractility abnormalities in a HFrEF cohort and assess whether insulin was a predictor of adverse outcomes in this entity.

**Methods:**

A total of 377 HFrEF patients who underwent cardiac MRI were included and divided according to diabetes status and the need for insulin treatment. LV structural and functional indices, as well as systolic strains, were measured. The determinants of impaired myocardial strains were assessed using linear regression analysis. The associated endpoints were determined using a multivariable Cox proportional hazards model.

**Results:**

T2DM patients on insulin displayed a higher indexed LV end-diastolic volume and LV mass than those with T2DM not on insulin or those without T2DM, despite similar LV ejection fractions, accompanied by a higher three-dimensional spherical index (P < 0.01). Worse longitudinal and circumferential peak systolic strain was shown to occur in T2DM patients on insulin (P < 0.01). Insulin treatment was independently associated with impaired magnitudes of systolic strain. The median follow-up duration was 32.4 months (IQR, 15.6–43.2 months). Insulin treatment remained consistently associated with poor outcomes after adjustment for established confounders, with an adjusted hazard ratio of 3.11; (95% CI, 1.45–6.87; P = 0.009) in the overall cohort and 2.16 (95% CI, 1.08–4.59; P = 0.030) in the diabetes cohort.

**Conclusions:**

Insulin may further lead to adverse LV remodeling and contractile dysfunction in the context of HFrEF with T2DM. Considerable care should be taken when treating HFrEF patients with insulin.

## Introduction

Heart failure (HF) has now become a global health burden, affecting an estimated 23 million people [1]. The clinical outcome of HF is still poor, and optimal treatment for both HF and its common comorbidities is equally important to lower the risk of hospitalization or death [2]. HF and type 2 diabetes mellitus (T2DM) often occur concomitantly, and both HF and T2DM closely interact with each other. The onset of one disease promotes a worse prognosis and further disease progression in the other [3]. Although the benefits of standard treatments are similar in HF patients irrespective of T2DM status, controversy remains about how to safely achieve and maintain glycemic control in HF patients with T2DM [3–5].

As monotherapy or in combination with other glycemic agents, insulin is a commonly used effective treatment for T2DM to achieve glycemic control. It alters renal handling of sodium, reenforcing fluid retention and triggering hyperinsulinemic hypoglycemia which might lead to myocardial ischemia and tachycardia by activating the sympathetic nervous system [5, 6]. Previous data from several registries and multicenter studies have demonstrated the association between insulin use and adverse outcomes in patients with chronic HF and T2DM, suggesting the detrimental effect of insulin on left ventricular (LV) remodeling in patients with HF comorbid with T2DM [2, 7–11].

Given that heart failure with reduced ejection fraction (HFrEF) accounts for approximately 50% of cases in the general HF population and is increasing in prevalence in the aging population, it is necessary to specifically clarify the adverse LV remodeling and mechanical alterations in patients with HFrEF and T2DM who take insulin [12]. However, to the best of our knowledge, little is known about the effect of insulin treatment on LV remodeling or on contractility abnormalities in this entity. Cardiac MRI has now been acknowledged as the optimal imaging method for comprehensive assessment of cardiac geometry and myocardial mechanics. Accordingly, the present study was designed to investigate the potential role of insulin use on LV remodeling and contractile abnormalities by cardiac MRI and to assess whether this therapy is associated with poor outcomes in patients with comorbid HFrEF and T2DM.

## Methods

### Data collection and study design

We performed an observational study to investigate the association between insulin treatment and outcome in patients with T2DM who develop HFrEF. The diagnosis of HFrEF was made according to the guidelines from the European Society of Cardiology (2021) [1]. Patients were initially enrolled between January 2015 and December 2021 via a hospital admission or consultation at an outpatient clinic with the presence of symptoms and/or signs of HF, an elevated amino-terminal pro-B-type natriuretic peptide (NT-proBNP), and a reduced LV ejection fraction (LVEF ≤ 40%) on cardiac MRI. Subjects who were 18 years old or under or had acute coronary syndrome, severe arrhythmia, or incomplete MRI images were excluded. The overall study cohort was further stratified by diabetes status and the need for insulin treatment: (1) the non-DM group; (2) the T2DM group without insulin treatment; and (3) the T2DM group with insulin treatment. Demographics, clinical characteristics, and laboratory measurements at baseline were collected from electronic clinical records. All patients were followed for the primary composite outcome of HF readmission, all-cause mortality and heart transplantation. Follow-up data were obtained from electronic medical records or phone calls to patients or family members. Follow-up duration was calculated as either the time from cardiac MRI to the occurrence of any endpoint or June 2022 (the last follow-up date).

This study was approved by the Biomedical Research Ethics Committees of our hospital and was complied with the Declaration of Helsinki. Written informed consent was waived because of the retrospective nature of the study. All medical data were protected with full confidentiality and used only for the purpose of the present study.

### MRI protocol and imaging postprocessing

MRI was performed on a 3-Tesla scanner (MAGNETOM Skyra/Tim Trio; Siemens Healthcare, Erlangen, Germany). Cine imaging was performed in the two-chamber long-axis view, four-chamber long-axis view, and 8–12 contiguous slices in the two-chamber short-axis view from the mitral valve to the LV apex using a retrospectively segmented ECG-gated balance steady state free precession sequence with the following parameters: repetition time = 2.81 ms; echo time = 1.22 ms; slice thickness = 8.0 mm; flip angle = 40°/50°; acquisition matrix = 166 × 208 pixels; and field of view = 340 × 284 mm^2^. Twenty-five frames were reconstructed per breath-hold acquisition for cine images.

All images were analyzed using commercially available CVI^42^ software (Circle Cardiovascular Imaging, Inc., Calgary, Alberta, Canada) by two investigators (K.S. with 10 years and G.Z. with 8 years of cardiac MRI experience) who were blinded to each other’s findings. The interobserver reproducibility of measurement for the MRI indices was good, with intraclass correlation coefficients (ICCs) ranging from 0.90 to 0.93. In a series of short-axis images, endocardial and epicardial borders were manually drawn at the LV end-diastolic and end-systolic phases, respectively. LV function parameters, including EF, end-diastolic volume (EDV), end-systolic volume (ESV), and stroke volume (SV), were automatically calculated. LV mass (LVM) was assessed by measuring the area between the endocardial and epicardial borders in each of the short-axis slices. LV papillary muscles were included in the LVM but not in the LV volume. LV function parameters and LVM were indexed to body surface area [13]. LV length was measured at the LV end-diastolic phases in a four-chamber view of long-axis images. The three-dimensional spherical index (3D-SI) was calculated as previously described [14].

A stack of short-axis cine images combined with long-axis images were loaded into the feature-tracking module for LV systolic strain analysis. We delineated LV endocardial and epicardial borders at the end-diastolic phases (reference phase) of all cine images. The short-axis reference points were inserted at the upper and lower septal insertion of the LV for global analysis of strain. Then, the software automatically traced and drew the contours throughout the remaining cardiac cycle. During the systolic phase, the LV shortens in the longitudinal and circumferential directions, causing negative global longitudinal peak strain (PS) and circumferential PS, whereas thickening in the radial direction causes positive global radial PS [15].

### Statistical analysis

Statistical analyses were performed using SPSS (IBM SPSS Inc., Armonk, New York, USA) and Prism (GraphPad software Inc., San Diego, California, USA). The normality of the data was determined using the Shapiro–Wilk test. Data are expressed as means with standard deviations or medians with interquartile ranges (IQRs) for continuous variables and frequencies for categorical variables. Clinical and MRI variables were compared among the non-DM, DM not on insulin, and DM on insulin subgroups using one-way analysis of variance, followed by the Bonferroni post hoc test, Kruskal–Wallis test, or chi-square test (Fisher’s exact test), as appropriate. The determinants of impaired LV contractile function were assessed separately in the overall cohort and in the T2DM cohort using linear regression analysis. Candidate variables with P < 0.10 in the univariable analysis and absence of collinearity were included in building the multivariable models. Long-term adverse outcomes were assessed using Kaplan–Meier survival analysis and compared among the groups using the log-rank test. The variables associated with adverse outcomes were determined using a multivariable Cox proportional hazards model. Variables with P < 0.10 in univariable analysis were included as cofactors for the Cox adjustment. Differences with a two-tailed P value < 0.05 were considered indicative of significance.

## Results

### Baseline characteristics of the study population

In general, among the 377 patients with HFrEF finally included in this study, 62.1% had T2DM, with one third of them prescribed with insulin. Table 1 depicts the baseline characteristics of the study population according to diabetes status and insulin use. T2DM patients with and without insulin treatment had a higher body mass index (P = 0.005) and systolic blood pressure (P < 0.001) than those without T2DM. T2DM patients who used insulin tended to be older than T2DM patients who did not use insulin and patients without T2DM (P = 0.076). However, sex, diastolic blood pressure, heart rate, and history of smoking and drinking were similar among the three groups, irrespective of their diabetes status and whether they were prescribed insulin (all P > 0.05).

**Table 1:** Baseline characteristics of the study population according to diabetes status and insulin use

	Non-DM (n = 143)	Non-insulin-treated DM (n = 156)	Insulin-treated DM (n = 78)
Age, yrs	55.3 ± 12.1	56.3 ± 12.5	59.1 ± 10.2
Male, n (%)	94 (65.7)	110 (70.5)	53 (67.9)
BMI, kg/m^2^	23.5 ± 4.1	24.9 ± 3.7*	24.9 ± 3.5*
SBP, mmHg	113.5 ± 21.8	120.4 ± 20.1*	128.8 ± 22.9*†
DBP, mmHg	73.6 ± 13.5	79.1 ± 14.0	78.2 ± 13.5
h, beats/min	86.1 ± 18.5	87.4 ± 18.7	85.4 ± 12.8
Smoking, n (%)	62 (43.4)	71 (45.5)	41 (52.6)
Drinking, n (%)	40 (28.0)	53 (34.0)	25 (35.9)
HF duration, n (%)			
≤ 1 yr	78 (54.5)	82 (52.6)	42 (53.8)
> 1 and ≤ 5 yrs	41 (28.7)	45 (28.8)	20 (25.6)
> 5 yrs	24 (16.8)	29 (18.6)	16 (20.5)
DM durarion, n (%)			
≤ 1 yr	NA	98 (62.8)	20 (25.6) λ
> 1 and ≤ 5 yrs	NA	23 (14.7)	11 (14.1)
> 5 yrs	NA	35 (22.4)	47 (60.3) λ
NYHA functional class III– IV, n (%)	105 (73.4)	118 (75.6)	64 (82.1)
Ischemic etiology of HF	48 (33.6)	47 (30.1)	30 (38.5)
Medical history, n (%)			
HT	35 (24.5)	71 (45.5) §	45 (57.7) §
AF	21 (14.7)	39 (25.0)	14 (17.9)
Dyslipidemia	28 (19.6)	59 (37.8) §	27 (34.6) §
COPD	16 (11.2)	13 (8.3)	10 (12.8)
SAS	5 (3.5)	9 (5.8)	4 (5.1)
Laboratory measurements			
NT‑proBNP, pg/mL	2527 (997, 5392)	2587 (1223, 6288)	3495 (1713, 10,059) &#
eGFR, mL/min/1.73m^2^	79.2 ± 23.4	77.6 ± 21.6	63.4 ± 28.9*†
TPN-T ng/L	22 (12.3, 46.6)	28.4 (16.5, 54.3)	41.3 (23.9, 73.6) &#
FBG, mmol/L	5.3 (4.8, 5.9)	7.2 (6.2, 8.9) &	9.5 (6.6, 13.1) &#
HbA1C, %	5.8 (5.6, 6.1)	6.9 (6.3, 7.7) &	7.9 (7.1, 9.6) &#
TG, mmol/L	1.3 ± 0.6	1.7 ± 0.9*	1.6 ± 0.3*
TC, mmol/L	4.0 ± 1.2	4.1 ± 1.2	3.9 ± 1.1
HDL‑C, mmol/L	1.1 ± 0.3	1.0 ± 0.3	1.1 ± 0.3
LDL‑C, mmol/L	2.4 ± 0.9	2.4 ± 0.9	2.2 ± 0.9
Hemoglobin, g/L	137 (126.5, 150)	137.5 (126, 154.3)	135.5 (106.5, 149)
Cardiovascular medications, n (%)			
Beta‑blocker	94 (65.7)	112 (71.8)	55 (70.5)
ACEI/ARB	91 (63.6)	116 (74.4)	53 (67.9)
Sacubitril/valsartan	32 (22.4)	37 (23.7)	20 (25.6)
Any diuretics	129 (90.2)	134 (85.9)	71 (90.0)
MRA	107 (74.8)	123 (78.8)	57 (73.1)
CCB	15 (10.5)	31 (19.9)	21 (26.9) §
Anti‑thrombotic agents	78 (54.5)	96 (61.5)	50 (64.1)
Statins	52 (36.4)	73 (46.8) §	46 (59.0) §λ
Digoxin	21 (14.7)	32 (20.5)	15 (19.2)

Data are presented as mean ± SD, media (Q1–Q3) or number (percentage)

One-way analysis of variance test: * P-value < 0.017 versus no DM cohort. † P-value < 0.017 versus DM not on insulin cohort. Kruskal-Wallis test: & P-value < 0.05 versus no DM cohort. # P-value < 0.05 versus DM not on insulin cohort. Chi-square test (Fisher’s exact test): § P-value < 0.05 versus no DM cohort. λ P-value < 0.05 versus DM not on insulin cohort

Abbreviations: DM, diabetes mellitus; BMI, body mass index; SBP, systolic blood pressure; DBP, diastolic blood pressure; HR, heart rate; HF, heart failure; NYHA, New York Heart Association; HT, hypertension; CAD, coronary artery disease; AF, atrial fibrillation; COPD, chronic obstructive pulmonary disease; SAS, sleep apnea syndrome;

NT-proBNP, amino-terminal pro-B-type natriuretic peptide; eGFR, estimated glomerular filtration rate; TPN-T, Troponin T; FBG, fasting blood glucose; HbA1C, glycated hemoglobin; TG, triglycerides; TC, cholesterol; HDL-C, high-density lipoprotein cholesterol content; LDL‑C, low-density lipoprotein cholesterol content. ACEI, angiotensin converting enzyme inhibitor; ARB, angiotensin receptor blocker; MRA, mineralocorticoid receptor antagonist; CCB, calcium-channel blocker

Compared to non-DM patients, T2DM patients with and without insulin treatment had a higher prevalence of hypertension (HT) (P < 0.001) and dyslipidemia (P = 0.002). However, ischemic etiology of HF was comparable among the three groups (P > 0.05). Insulin-requiring patients had the highest levels of NT-proBNP, troponin T, fasting blood glucose and glycated hemoglobin (HbA1C) but the lowest estimated glomerular filtration rate (eGFR) across groups (all P < 0.001). the use of cardiovascular medications was similar among the three groups, with the exception of calcium-channel blockers (P = 0.006) and statins (P = 0.005), which were more likely to be prescribed to T2DM patients who took insulin.

Additionally, between both groups of T2DM patients, individuals on insulin treatment had a longer diabetes duration than those without (P < 0.001). Moreover, no significant difference was found in the oral hypoglycemic medications used between the two groups (all P > 0.05; Table 2).

**Table 2:** Oral hypoglycemic treatment in the diabetic cohort

	DM treatment without insulin (n = 156)	DM treatment with insulin (n = 78)	P-value
Metformin	58 (37.2)	25 (32.1)	P = 0.440
Sulfonylureas	31 (19.9)	17 (21.8)	P = 0.731
α-Glucosidase inhibitors	41 (26.3)	21 (26.9)	P = 0.917
SGLT-2	47 (30.1)	32 (41.0)	P = 0.097
DPP-4 inhibitors	10 (6.4)	8 (10.3)	P = 0.298

Data are presented as number (percentage)

Abbreviations: DM, diabetes mellitus; α-GI, α-Glucosidase inhibitors; SGLT-2, sodium-glucose cotransporter-2 inhibitors; DPP-4 inhibitors, dipeptidyl peptidase 4 inhibitors

### Association of insulin with LV remodeling and abnormal myocardial mechanics

**Fig. 1:**
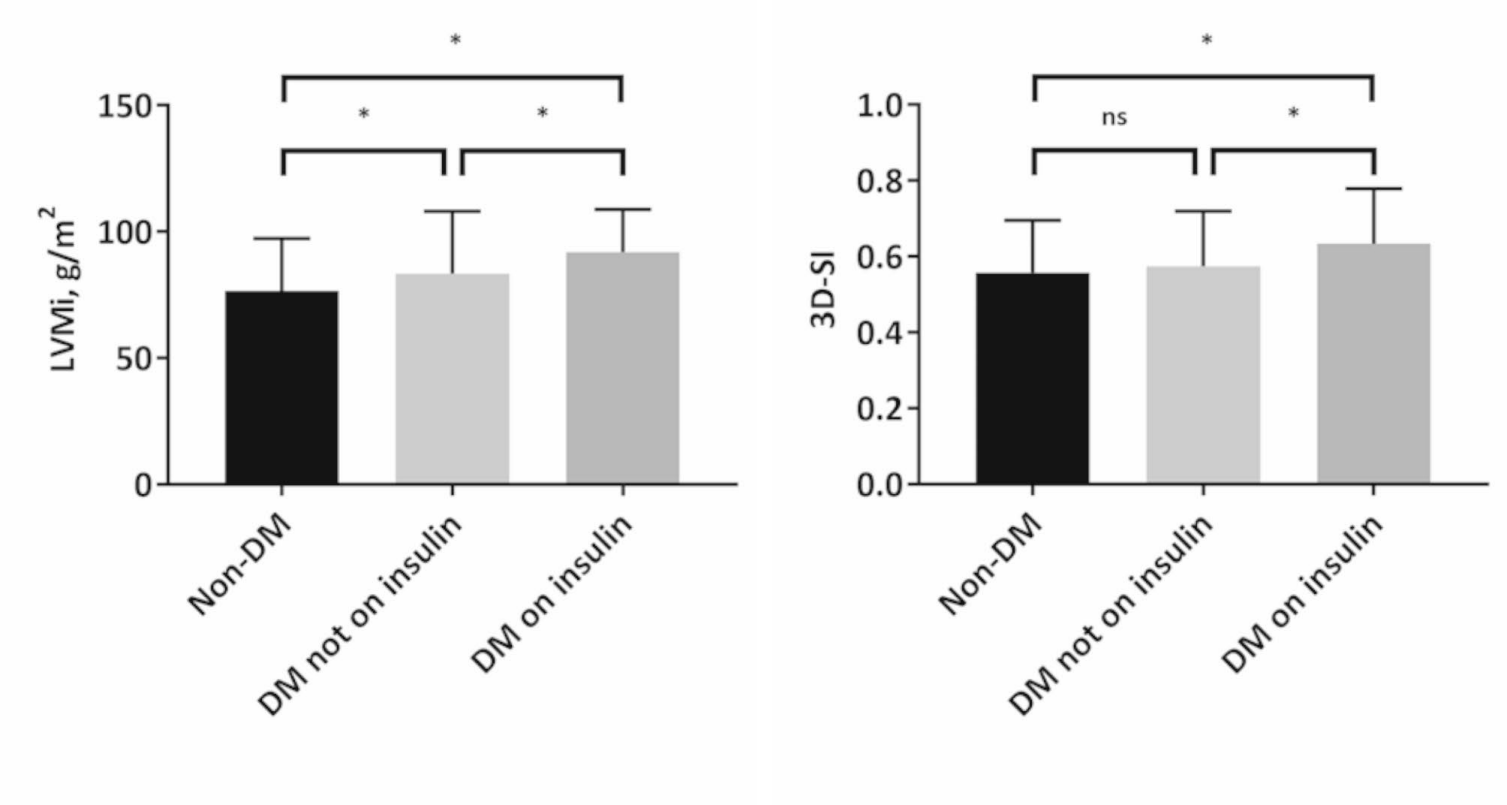
Differences of indexed left ventricular mass and 3D-SI across the groups. Abbreviations: LVMi, indexed left ventricular mass; 3D-SI, three-dimensional spherical index; DM, diabetes mellitus; ns, no statistical significance; *, P-value < 0.017

Although LVEF was similar across groups (P > 0.05), T2DM patients on insulin displayed higher LVEDV (P = 0.009), LVESV (P = 0.015), and LVSV (P = 0.019) than those with T2DM not on insulin and those without T2DM. However, when corrected for body size, these indices did not notably differ among the groups except for indexed LVEDV (DM on insulin: 173.6 ± 69.5 mL/m^2^ vs. DM not on insulin: 153.4 ± 48.8 mL/m^2^ vs. non-DM: 155.1 ± 42.6 mL/m^2^; P = 0.014). Moreover, LVM was greater in T2DM patients on insulin treatment than in those without insulin treatment, and LVM in both of these groups was greater than that in participants without T2DM (P < 0.001); the significant difference remained even when taking body size into account (DM on insulin: 92.7 ± 16.8 g/m^2^ vs. DM not on insulin: 83.5 ± 24.6 g/m^2^ vs. non-DM: 76.6 ± 20.7 g/m^2^; P = 0.005; Fig. 1). Finally, insulin-requiring subjects demonstrated a higher 3D-SI than diabetic subjects not taking insulin or those without diabetes (DM on insulin: 0.63 ± 0.15 vs. DM not on insulin: 0.57 ± 0.14 vs. non-DM: 0.55 ± 0.13; P = 0.001; Fig. 1). More details can be found in Table 3.

**Table 3:** LV geometry and function of the study population

	Non-DM (n = 143)	Non-insulin-treated DM (n = 156)	Insulin-treated DM (n = 78)
LVEDV, mL	259.1 (202.7, 320.5)	267.3 (197.2, 312.9)	282.2 (210.8, 351.5) &#
LVEDV index, mL/m^2^	155.1 ± 42.6	153.4 ± 48.8	173.6 ± 69.5*†
LVESV, mL	189.8 (137.1, 252.6)	193.8 (137.8, 247.1)	211.7 (149.7, 284.7) &#
LVESV index, mL/m^2^	117.7 ± 41.0	116.2 ± 45.9	131.3 ± 63.7
LVSV, mL	62.9 ± 22.9	61.5 ± 23.8	71.1 ± 26.9*†
LVSV index, mL/m^2^	37.4 ± 10.9	36.1 ± 10.1	40.9 ± 12.7
LVEF, %	25.7 ± 8.6	25.2 ± 9.0	25.2 ± 9.2
LVM, g	127.4 ± 37.1	145.4 ± 39.8*	164.2 ± 36.2*†
LVM index, g/m^2^	76.6 ± 20.7	83.5 ± 24.6*	92.7 ± 16.8*†
LVEDD, mm	64.3 ± 6.7	64.7 ± 6.1	65.2 ± 6.3
LVESD, mm	51.3 ± 4.4	52.0 ± 5.6	50.5 ± 5.9
LV length, mm	96.7 ± 12.1	97.3 ± 11.2	100.2 ± 10.8
3D-SI	0.55 ± 0.13	0.57 ± 0.14	0.63 ± 0.15*†

Data are presented as mean ± SD, or media (Q1–Q3).

One-way analysis of variance test: * P-value < 0.017 versus no DM cohort. † P-value < 0.017 versus DM not on insulin cohort. Kruskal-Wallis test: & P-value < 0.05 versus no DM cohort. # P-value < 0.05 versus DM not on insulin cohort

Abbreviations: LV, left ventricular; DM, diabetes mellitus; LVEDV, left ventricular end-diastolic volume; LVESV, left ventricular end-systolic volume; LVSV, left ventricular stroke volume; LVEF, left ventricular ejection fraction; LVM, left ventricular mass; LVEDD, left ventricular end-diastolic dimension; LVESD, left ventricular end-systolic dimension; 3D-SI, three-dimensional spherical index

**Fig. 2:**
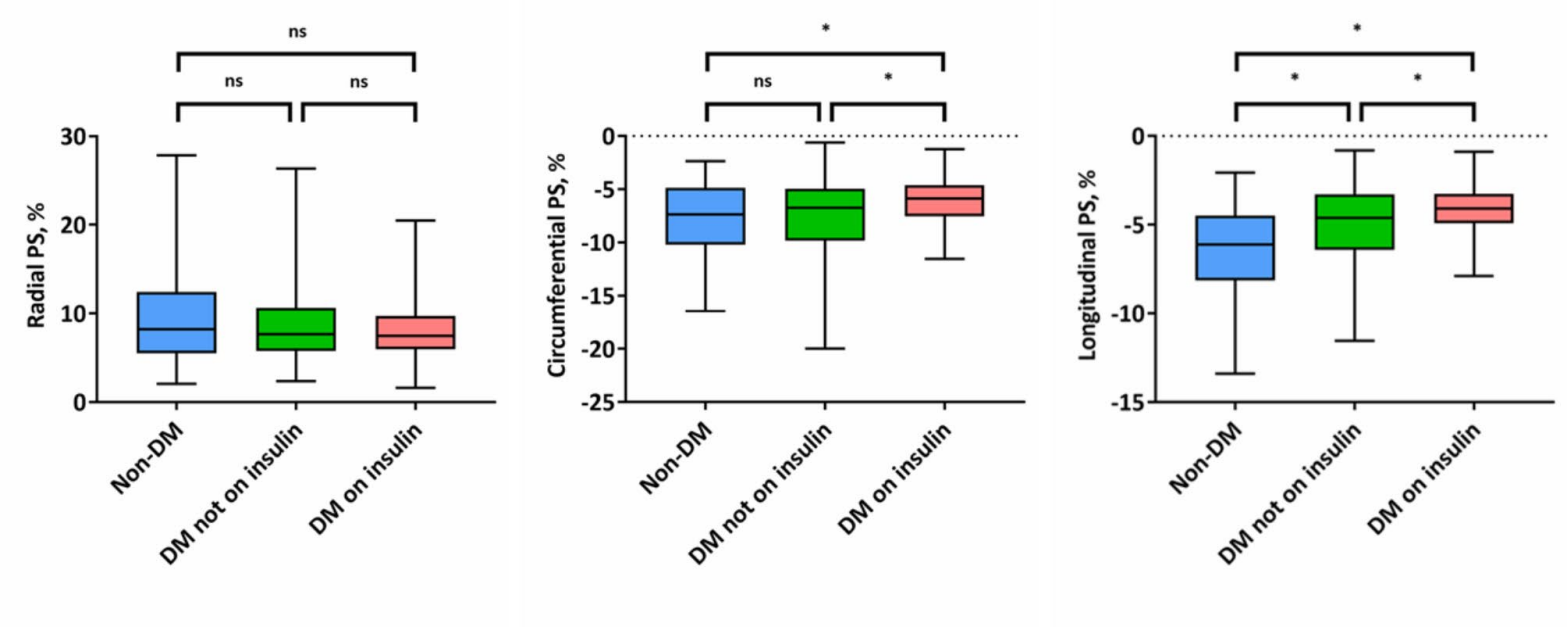
Differences of magnitude of global left ventricular systolic PS across the groups. Abbreviations: DM, diabetes mellitus; PS, peak strain; ns, no statistical significance; *, P-value < 0.017

As shown in Fig. 2, deterioration of the magnitude of LV global longitudinal PS was greatest in insulin-treated patients with T2DM, intermediate in non-insulin-treated patients with T2DM, and lowest in non-DM patients (DM on insulin: -4.2% ± 1.5% vs. DM not on insulin: -5.0% ± 2.2% vs. non-DM: -6.4% ± 2.4%; P < 0.001). T2DM patients on insulin treatment exhibited more severe impairment in the magnitude of LV global circumferential PS than T2DM patients not taking insulin or patients without T2DM (DM on insulin: -6.1% ± 2.2% vs. DM not on insulin: -7.3% ± 3.2 vs. non-DM: -7.6% ± 3.3%; P = 0.007). Nevertheless, a nonsignificant trend was found for the magnitude of LV global radial PS across groups (DM on insulin: 9.5% ± 5.2% vs. DM not on insulin: 8.9% ± 4.5% vs. non-DM: 7.9% ± 3.3%; P = 0.104).

Univariable linear regression analysis of the whole study population showed that obesity; the presence of HT, coronary artery disease and T2DM; insulin use; NT-proBNP; eGFR and indexed LVM were associated with impaired longitudinal PS (all P < 0.05). After multivariable adjustment, the presence of T2DM and insulin use remained independent determinants of impaired longitudinal PS (T2DM: multivariable β = 0.18, P < 0.01; insulin use: multivariable β = 0.13, P < 0.05). Likewise, a similar association was observed for circumferential PS when adjusted for the same variables (T2DM: multivariable β = 0.10, P < 0.05; insulin use: multivariable β = 0.16, P < 0.01) (Table 4).

**Table 4:** Linear regression analysis of the determination of impaired LV contractile function in overall cohort

	Circumferential PS		Longitudinal PS	
	Univariable β	Multivariable β	Univariable β	Multivariable β
Age, per 10 units increase	0.06		0.02	
Obesity#, yes or no	0.18†	0.20†	0.20†	0.12*
HT, yes or no	0.04		0.22†	
CAD, yes or no	0.07		0.19†	0.15†
T2DM, yes or no	0.12*	0.10*	0.34†	0.18†
Insulin use, yes or no	0.20†	0.16†	0.28†	0.13*
NT-proBNP§, per 1 unit increase	0.20†	0.19†	0.23†	0.17†
eGFR, per 1 unit increase	-0.06		-0.11*	
Indexed LVM, per 1 unit increase	0.20†	0.16†	0.26†	0.18†

β is adjusted regression coefficient

* P-value < 0.05. † P-value < 0.01

# Subjects with body mass index ≥ 25 kg/m^2^ were classified as obese group that proposed by the World Health Organization for Asian populations

§ NT-proBNP is log-transformed before being included in the regression analysis

Abbreviations: LV, left ventricular; PS, peak strain; HT, hypertension; CAD, coronary artery disease; DM, diabetes mellitus; NT-proBNP, amino-terminal pro-B-type natriuretic peptide; eGFR, estimated glomerular filtration rate; LVM, left ventricular mass

### Association between insulin treatment and adverse outcomes

**Fig. 3:**
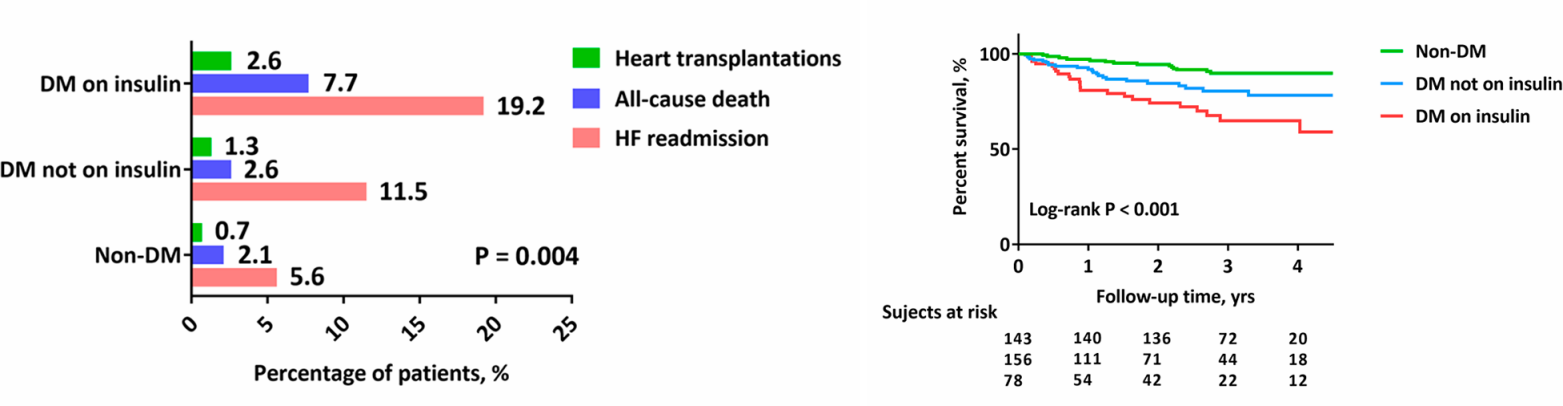
Prevalence of adverse outcomes and survival curves during a median follow-up of 2.7 years . Abbreviations: HF, heart failure; DM, diabetes mellitus

The entire study cohort was observed for a median of 32.4 months (IQR, 15.6–43.2 months). During the follow-up period, the primary composite outcome, which included 41 HF readmissions (10.9%), 13 all-cause deaths (3.4%) and 5 heart transplantations (1.3%), occurred in 59 patients (15.6%). The proportion of patients experiencing an adverse event was significantly higher in the T2DM group that received insulin than in either the T2DM group that did not receive insulin or the non-DM group (29.5% vs. 15.4% vs. 8.4%; P = 0.004). Detailed event data in the three groups are shown in Fig. 3. By Kaplan–Meier survival analysis, T2DM patients prescribed insulin showed worse long-term outcomes during follow-up (log-rank P < 0.001; Fig. 3).

On Cox multivariable analysis, T2DM patients prescribed insulin had a 3-fold increase in the risk of primary composite outcome compared with non-DM patients (HR = 3.11; 95% CI, 1.45–6.87; P = 0.009), whereas T2DM patients not prescribed insulin had a 2-fold increase (HR = 2.16; 95% CI, 1.06–4.40; P = 0.015) after adjustment for baseline characteristics, etiology of HF, NT-proBNP, eGFR, and utility of sacubitril/valsartan. (Table 5). Of note, when we performed Cox multivariable analysis separately in the T2DM group, insulin treatment remained associated with the primary composite outcome compared to those not treated with insulin (HR = 2.85; 95% CI, 1.40–5.51; P = 0.007) after adjustment for the abovementioned prognostic variables. Furthermore, despite the attenuated risk, the major confounders HbA1C and DM duration did not change the relationship between insulin treatment and adverse outcomes, with an adjusted HR of 2.16 (95% CI, 1.08–4.59; P = 0.030) (Table 6).

**Table 5:** Cox proportional hazards regression analysis to identify the association of DM status and insulin use with adverse outcomes in overall cohort

	HR (95% CI)	P-value
Unadjusted model		
Non-DM	1.00 (reference)	
Non-insulin-treated DM	2.82 (1.42–5.61)	P = 0.003
Insulin-treated DM	4.83 (2.39–9.74)	P < 0.001
Model 1: adjusted for age, sex, BMI* and NYHA function class		
Non-DM	1.00 (reference)	
Non-insulin-treated DM	2.59 (1.29–5.21)	P = 0.008
Insulin-treated DM	4.41 (2.14–9.07)	P < 0.001
Model 2: adjusted for model 1 combined with etiology of HF		
Non-DM	1.00 (reference)	
Non-insulin-treated DM	2.58 (1.28–5.20)	P = 0.007
Insulin-treated DM	4.45 (2.16–9.15)	P < 0.001
Model 3: adjusted for model 2 combined with NT-proBNP§, eGFR, and use of sacubitril/valsartan		
Non-DM	1.00 (reference)	
Non-insulin-treated DM	2.16 (1.06–4.40)	P = 0.015
Insulin-treated DM	3.11 (1.45–6.87)	P = 0.009

* BMI is considered as binary variable (BMI ≥ 25 kg/m^2^) when including in the Cox model

§ NT-proBNP is log-transformed before being included in the Cox model

Abbreviations: DM, diabetes mellitus; HR, hazards ratio; CI, confidence interval; BMI, body mass index; NYHA, New York Heart Association; NT-proBNP, amino-terminal pro-B-type natriuretic peptide; eGFR, estimated glomerular filtration rate

**Table 6:** Cox proportional hazards regression analysis to identify the association between insulin use and adverse outcomes in DM cohort

	HR (95% CI)	P-value
Unadjusted model		
Non-insulin-treated DM	1.00 (reference)	
Insulin-treated DM	3.01 (1.66–5.44)	P < 0.001
Model 1: adjusted for age, sex, BMI* and NYHA function class		
Non-insulin-treated DM	1.00 (reference)	
Insulin-treated DM	2.94 (1.62–5.33)	P < 0.001
Model 2: adjusted for model 1 combined with etiology of HF		
Non-insulin-treated DM	1.00 (reference)	
Insulin-treated DM	2.93 (1.61–5.34)	P < 0.001
Model 3: adjusted for model 2 combined with NT-proBNP§, eGFR, and use of sacubitril/valsartan		
Non-insulin-treated DM	1.00 (reference)	
Insulin-treated DM	2.85 (1.40–5.51)	P = 0.007
Model 4: adjusted for model 3 combined with HbA1C and DM duration		
Non-insulin-treated DM	1.00 (reference)	
Insulin-treated DM	2.16 (1.08–4.59)	P = 0.030

* BMI is considered as binary variable (BMI ≥ 25 kg/m^2^) when including in the Cox model

§ NT-proBNP is log-transformed before being included in the Cox model

Abbreviations as in Table 5

## Discussion

The present study highlights evidence supporting the need to consider the safety of insulin therapy before prescribing it to patients with HFrEF. The main findings were as follows: (1) insulin usage in comorbid HFrEF and T2DM seems to be associated with increased LV volume, aggravated LVH and spherical-like LV remodeling. (2) Despite a similar LVEF across groups, T2DM patients who took insulin displayed deteriorated LV contractility, with worse systolic PS in the longitudinal and circumferential directions. Insulin treatment was the independent determinant of the reduced magnitude of global longitudinal and circumferential PS. (3) Finally, insulin treatment was found to be associated with an increased risk of adverse clinical outcomes in patients with HFrEF, and this association persisted regardless of DM status and duration.

### Role of insulin in the development of adverse LV remodeling

Insulin has long been used as a second-line therapy to manage T2DM when oral hypoglycemic drugs fail to achieve the targeting glycemic level. Insulin usage among high-risk patients has the advantage of protecting against glucotoxicity that may lead to diabetes-related microvascular complications [6, 11]. However, one of the potential adverse effects of insulin in HF is that it contributes to sodium and water retention, which may exacerbate cardiac decompensation [16]. Our study, which included MRI assessment, showed that insulin treatment is associated with further LV dilatation and spherical-like remodeling in the setting of T2DM. The current findings could in turn explain the observations by Cosmi et al., who reported that insulin-treated patients in an HFrEF cohort suffered from more signs of cardiac congestion as well as a more advanced NYHA functional class [10]. Thus, our study indicated a simple and effective MRI parameter that may be used as a marker of congestion and predictor of poor outcomes.

A more recent study reported that patients with comorbid HFrEF and T2DM exhibited higher LVM than nondiabetic patients [17]. Nevertheless, little is known about the reciprocal relationship between insulin treatment and LVM in HFrEF patients with T2DM. The present study extends this cardiac hypertrophy to a specific population in which patients with concomitant HFrEF and T2DM are prescribed insulin. We speculate that insulin itself could induce cardiomyocyte hypertrophy alongside myocardial fibrosis and collagen accumulation [18]. Whether the observed LVH in insulin-treated patients is reversible deserves further investigation.

### Insulin use and LV contractile dysfunction

To the best of our knowledge, our study was the first to use strain analyses derived from MRI feature tracking to explore the effect of insulin use on changes in LV mechanics. In a CHS cohort study, Garg et al. observed that higher insulin secretion was associated with worse LV longitudinal systolic function in an older population with prediabetes and diabetes [19]. In contrast, with regard to HFrEF comorbid with T2DM, data from this study displayed worse longitudinal and circumferential systolic strain values in individuals on insulin than in those not on insulin. More importantly, after considering the established variables that are linked to the magnitude of myocardial strain, insulin treatment remained associated with reduced systolic strain in the multivariable regression model in the overall cohort. This finding may reflect that insulin is detrimental to cardiomyocytes in HFrEF patients in the context of T2DM. Supporting evidence reveals that in addition to water and sodium retention, insulin-induced hypoglycemia could trigger widespread abnormal autonomic nervous system activation, systemic inflammation, oxidative stress, and a prothrombotic state, resulting in myocardial ischemia, overexpression of sodium-hydrogen exchangers, and microvasculature disturbance, thereby promoting cardiomyocyte injury and dysfunction [20–22].

### Insulin treatment and adverse clinical outcomes

The relationship between insulin uses and adverse outcomes is not fully understood. In the context of prediabetes and established T2DM, analyses from large-scale clinical trials (ORIGIN and DEVOTE) of cardiovascular events in recent years revealed that the use of insulin did not lead to major cardiovascular outcomes or to increased HF hospitalization and HF recurrence [23–25]. Unfortunately, those subjects with advanced NYHA function class (III or IV) were not enrolled in these trials. Conversely, for HF, several observational studies reported an increased rate of adverse outcomes related to insulin therapy in patients with concurrent T2DM and HF [2, 7–11]. Indeed, since T2DM patients with insulin therapy generally have a longer duration of diabetes, undesirable glycemic control, and a higher risk of cardiovascular comorbidities and are often treated with insulin in combination with other oral antidiabetic therapies, these factors may be the major confounders in assessing the association between insulin use and adverse outcomes. More than that, the use of some specific medications for HF, such as sacubitril/valsartan, show evidence of providing cardiovascular benefits [26]. Instead, our study attempted to correct for the potential bias as much as possible. We found that even after adjustment for the duration of T2DM and level of glycemic control, the use of insulin in HFrEF patents to treat diabetes still led to a 2-fold increase in the risk of the primary composite outcome, indicating that the negative prognostic effect of insulin is unlikely to be changed. Insulin should be used with caution in the treatment of comorbid HFrEF and T2DM. Other alternative agents are preferred if adequate glycemic control can be achieved.

### Study limitations

Several limitations have to be acknowledged in this study. First, due to the relatively small sample size of insulin-dependent patients and the adherence uncertainty of patients during the follow-up period, the usage and type of insulin were not considered in the current study. Further randomized trials should be conducted to address whether the dose or type of insulin is associated with LV systolic dysfunction. Second, we did not perform quantitative measurement of myocardial fibrosis in the study cohort. It would be interesting to explore the effect of insulin on the development of myocardial fibrosis using late gadolinium enhancement or extracellular volume derived from cardiac MRI. Third, since the incidence of all-cause death and heart transplantations in our cohort was low, we used a composite outcome to enhance the statistical power in our Cox multivariable analysis model. It is necessary to estimate the association between the use of insulin and risk of each clinical outcome. Finally, we must acknowledge the retrospective nature of this study and selecting bias is inevitable.

In conclusion, our study showed evidence of detrimental effects of insulin on adverse LV remodeling as well as systolic contractility in HFrEF patients with T2DM. In the context of HFrEF, the use of insulin to treat diabetes is associated with a higher risk of adverse outcomes, regardless of the greater severity of diabetes. Specific management strategies and close monitoring are needed when considering insulin treatment in patients with comorbid HFrEF and T2DM.

## Data Availability

The datasets used and/or analyzed during the current study are available from the corresponding author on reasonable request.

## List of abbreviations

HF: Heart failure
T2DM: Type 2 diabetes mellitus
LV: Left ventricular
HFrEF: Heart failure with reduced ejection fraction
NT-proBNP: Amino-terminal pro-B-type natriuretic peptide
ICCs: Intraclass correlation coefficients
EF: Ejection fraction
EDV: End-diastolic volume
ESV: End-systolic volume
LVM: Left ventricular mass
SV: Stroke volume
3D-SI: Three-dimensional spherical index
PS: Peak strain
HT: Hypertension
HbA1C: Glycated hemoglobin
eGFR: Estimated glomerular filtration rate
